# Dermatophyte Resistance to Antifungal Drugs: Mechanisms and Prospectus

**DOI:** 10.3389/fmicb.2018.01108

**Published:** 2018-05-29

**Authors:** Nilce M. Martinez-Rossi, Tamires A. Bitencourt, Nalu T. A. Peres, Elza A. S. Lang, Eriston V. Gomes, Natalia R. Quaresemin, Maíra P. Martins, Lucia Lopes, Antonio Rossi

**Affiliations:** ^1^Department of Genetics, Ribeirão Preto Medical School, University of São Paulo, Ribeirão Preto, Brazil; ^2^Department of Morphology, Federal University of Sergipe, Aracaju, Brazil

**Keywords:** dermatophyte, stress response, drug target, drug resistance, natural compounds, kinase, Hsp

## Abstract

Dermatophytes comprise pathogenic fungi that have a high affinity for the keratinized structures present in nails, skin, and hair, causing superficial infections known as dermatophytosis. A reasonable number of antifungal drugs currently exist on the pharmaceutical market to control mycoses; however, their cellular targets are restricted, and fungi may exhibit tolerance or resistance to these agents. For example, the stress caused by antifungal and cytotoxic drugs in sub-inhibitory concentrations promotes compensatory stress responses, with the over-expression of genes involved in cellular detoxification, drug efflux, and signaling pathways being among the various mechanisms that may contribute to drug tolerance. In addition, the ATP-binding cassette transporters in dermatophytes that are responsible for cellular efflux can act synergistically, allowing one to compensate for the absence of the other, revealing the complexity of drug tolerance phenomena. Moreover, mutations in genes coding for target enzymes could lead to substitutions in amino acids involved in the binding of antifungal agents, hindering their performance and leading to treatment failure. The relevance of each one of these mechanisms of resistance to fungal survival is hard to define, mainly because they can act simultaneously in the cell. However, an understanding of the molecular mechanisms involved in the resistance/tolerance processes, the identification of new antifungal targets, as well as the prospective of new antifungal compounds among natural or synthetic products, are expected to bring advances and new insights that facilitate the improvement or development of novel strategies for antifungal therapy.

## Introduction

The growing prevalence of human fungal infections, especially in immunocompromised patients, has resulted in these diseases becoming a worldwide public health issue. The immune status of the host determines the outcome of the disease, which may range from limited cutaneous or subcutaneous to invasive, disseminated, and life-threatening infections (Kohler et al., 2017). The most common fungal infections comprise the dermatomycoses, which constitute fungal infections in the skin that affect both immunocompetent and immunocompromised individuals. Yeasts from the genera *Candida* and *Malassezia*, and molds such as the dermatophytes, serve as the most prevalent etiologic agents of these infections (Havlickova et al., 2008). Dermatophytes comprise a class of primary pathogenic fungi; i.e., not opportunistic, that requires the cleavage of keratin found in the skin, hair, and nails to acquire nutrients for their survival. The secretion of a broad spectrum of lytic enzymes, such as lipases and proteases, especially keratinases, by the fungal hyphae represents their most studied virulence factor, allowing fungal colonization and maintenance in the host tissue (Monod, 2008; Peres et al., 2010a).

Dermatophytes can be classified as zoophilic or anthropophilic depending on their host prevalence. Whereas zoophilic species are able to cause infections in both animals and humans, the anthropophilic dermatophytes are only able to infect humans. This classification is important as the clinical manifestation is highly influenced by the fungal species along with the immune response they trigger during infection. In humans, zoophilic species are responsible for acute infections, mostly owing to the intense induction of the inflammatory response in the infected tissue. In comparison, anthropophilic species are associated with chronic infections, with a lower cellular infiltration in the skin (Hube et al., 2015). In the first stages of infection, dermatophytes colonize the skin and stimulate keratinocytes to produce different patterns of cytokines that mediate the inflammatory response and accumulation of leukocytes, mainly neutrophils, in the infected tissue (Peres et al., 2010b; Hube et al., 2015; Martinez-Rossi et al., 2017). Dermatophytes are initially recognized by receptors in the host innate immune cells, such as macrophages, neutrophils, and dendritic cells, which induce the activation of an adaptive response to control infection. *In vitro* and *in vivo* models of infection have shown that anthropophilic species (including *Trichophyton tonsurans*) induce keratinocytes to secrete a limited spectrum of cytokines, mainly IL-8, IL-6, and IL-1β. Alternatively, the zoophilic species *Arthroderma benhamiae* induces a broader spectrum of cytokines, such as IL-8, Il-6, IL-1β, IL-10, IL-2, IL-15, TGF-β, contributing to the higher amount of inflammatory cells in the infection site, which is responsible for fungal clearance, healing, and tissue remodeling (Hau et al., 2015).

Induction of the adaptive immunity by innate cells triggers both Th1 and Th17 responses, which are important to control dermatophyte infection, by stimulating cellular-mediated immunity (Hau et al., 2015; Heinen et al., 2016). Th17 response increases the infiltration of neutrophils in the infection site, in turn promoting the activation of epithelial cells to produce chemotactic molecules and antimicrobial peptides (Heinen et al., 2016). Furthermore, the production of antibodies and complement activation is also important to control dermatophytes. Serum analysis of patients with dermatophytosis shows high levels of Th2 cytokines, such as IL-4, IL-5, and IgE (Woodfolk and Platts-Mills, 2001; Hau et al., 2015; Gupta C. et al., 2016). The Th1, Th2, and Th17 immune responses observed in dermatophytosis is believed to reflect the different immunological status of the hosts (Hau et al., 2015) and may also be due to the infecting species. Moreover, genetic factors play a further role in the susceptibility to invasive dermatophytosis (Garcia-Romero and Arenas, 2015). In particular, mutations in the *card9* gene render a more invasive lesion in patients with dermatophytosis, together with a concomitant lower level of IL17 (Lanternier et al., 2013).

Despite the availability of several antifungal drugs for clinical use, these act on a limited number of cellular targets. Commercially available drugs act on the plasma membrane, cell wall, nucleic acids, and the process of cell division. In particular, three classes of antifungal drugs interfere with the plasma membrane. Azoles and triazoles inhibit the enzyme sterol 14 α-demethylase, and the allylamines inhibit squalene epoxidase, both leading to inhibition of ergosterol biosynthesis. Allylamines also lead to the accumulation of lanosterol, a toxic intermediary compound of the ergosterol biosynthesis pathway. Polyenes bind to ergosterol, thus reducing plasma membrane integrity. Echinocandins comprise a class of cell wall interfering agents that inhibit β-glucan synthase. The pyrimidine analog 5-fluorocytosine inhibits DNA and RNA synthesis, leading to cell division and protein synthesis defects. Griseofulvin (GRS) interferes with microtubule formation, impairing fungal growth and cell division. Finally, ciclopirox oleamine functions as a protein synthesis inhibitor, which is often used against dermatophytes (Martinez-Rossi et al., 2008).

The overlapping mechanisms of action of the commonly used drugs may contribute to the multidrug resistance (MDR) phenotypes observed for several pathogenic fungi. Moreover, is common for patients to neglect and abandon treatment, owing to its requirement for long term use and the associated side effects (Martinez-Rossi et al., 2008; Peres et al., 2010a). In turn, clinical and/or microbiological resistance may result in treatment failure. Specifically, clinical resistance is defined as the failure of infection clearance even with the administration of drugs that display *in vitro* activity, whereas microbial resistance relies on different molecular mechanisms used by the fungus to overcome the inhibitory effects of the antifungal drugs (Kanafani and Perfect, 2008). Several such mechanisms may be triggered by the fungus, which can act alone or in combination. These mechanisms involve the overexpression of efflux pumps or detoxification enzymes, along with target and drug modification (Martinez-Rossi et al., 2008; Cowen et al., 2015; Pianalto and Alspaugh, 2016).

In this review, we provide a special emphasis on the mechanisms of stress response and antifungal resistance in dermatophytes, and the prospective for the use of novel drugs to treat cutaneous fungal infections.

## Natural and Synthetic Compounds with Antifungal Activity

Fungal infections are increasing owing to various factors, whereas the current antifungal arsenal shows considerable drawbacks mainly related to their side effects and the emergence of drug resistance (Martinez-Rossi et al., 2008). Therefore, it is imperative to develop new agents with effective and specific modes of action.

Currently, synthetic drugs acting on novel cellular targets are under investigation, or in preclinical or clinical trials. These drugs include inhibitors of metabolic pathways, such as the glyoxylate cycle, pyrimidine biosynthesis, cytochrome P450 pathway, iron metabolism, acetate metabolism, and heme biosynthesis, along with signal transduction pathways, such as mitogen-activated protein (MAP) kinase and calcium signaling pathways, as well as transcription factor and DNA-binding and histone deacetylase inhibitors, with the latter representing epigenetic therapy (McCarthy et al., 2017) (**Table 1**).

**Table 1:** Novel antifungal drugs currently under evaluation, preclinical or clinical trial.

Target	Drugs (phase of development)	Proposed mechanism of action	Fungi tested	Reference
Cell membrane	BHBM D0 (preclinical)	Interferes with fungal vesicles, and synthesis of sphingolipids	*Cryptococcus* spp. *Candida* spp. *Aspergillus fumigatus Rhizopus oryzae Blastomyces dermatitidis Histoplasma capsulatum Coccidioides Paecilomyces variotii Pneumocystis jirovecii*	Mor et al., 2015
	VT-1129 (clinical trial)	Inhibition of Cyp51 (lanosterol 14-α-demethylase)	*Cryptococcus Candida* sp.	Lockhart et al., 2016; Schell et al., 2017b
	VT-1598 (investigational)	Inhibition of Cyp51 (lanosterol 14-α-demethylase)	*Candida* sp. *Aspergillus* sp. *Cryptococcus* sp. *Rhizopus* sp. *Coccidioides* sp.	Wiederhold et al., 2018a,b
	VT-1161 (clinical trial)	Inhibition of Cyp51 (lanosterol 14-α-demethylase)	*Candida* sp. *Trichophyton mentagrophytes Trichophyton rubrum Epidermophyton floccosum*	Warrilow et al., 2014; Garvey et al., 2015; Schell et al., 2017b
Nucleic acid	F901318 (clinical trial)	Inhibition of dihydroorotate dehydrogenase (pyrimidine biosynthesis pathway)	*Aspergillus* sp.	Oliver et al., 2016
	MGCD290 (clinical trial)	Inhibition of the Hos2 histone deacetylase	*Candida* sp. *Aspergillus* sp. *Cryptococcus* sp. *Rhodotorula* sp. *Fusarium* sp. *Trichosporon* sp. *Scedosporium* sp. *Zygomycetes* sp.	Pfaller et al., 2009
Mitochondria	T-2307^3^ (clinical trial)	Disruption of the fungal mitochondrial membrane potential	*Candida* sp. *Cryptococcus* sp. *Aspergillus* sp. *Fusarium solani Malassezia furfur Mucor racemosus*	Mitsuyama et al., 2008; Nishikawa et al., 2017
	Ilicicolin H (investigational)	Inhibition of the cytochrome bc1 reductase	*Candida* sp. *Aspergillus* sp. *C. neoformans*	Singh et al., 2012
Heme biosynthesis	Sampangine (investigational)	Interferes with heme metabolism	*C. neoformans C. albicans C. glabrata C. krusei A. fumigatus*	Agarwal et al., 2008
	Hemofungin (investigational)	Inhibition of ferrochelatase, an enzyme from the heme biosynthesis pathway	*Aspergillus* sp. *Candida* sp. *Fusarium* sp. *Rhizopus* sp.	Ben Yaakov et al., 2016
Metabolism	AR-12 (clinical trial)	Inhibition of acetyl coenzyme A (acetyl-CoA) synthetase (acetate metabolism)	*C. neoformans Candida* sp. *Fusarium* sp. *Mucor* sp. *Blastomyces* sp. *Histoplasma* sp.	Koselny et al., 2016; Kushwaha et al., 2017
			*Coccidioides* sp. *Trichophyton rubrum*	
	Mohangamides (investigational)	Inhibition of the isocitrate lyase (glyoxylate pathway)	*Candida albicans*	Bae et al., 2015; McCarthy et al., 2017
	ASP2037	Unknown (iron metabolism)	*Aspergillus* sp.	Arendrup et al., 2016
Cell wall	Biafungin (CD101) (clinical trial)	Inhibition of glucan synthase	*Candida* sp. *Aspergillus* sp.	Pfaller et al., 2016
	Scy-078 (clinical trial)	Inhibition of glucan synthase	*Candida* sp. *Aspergillus* sp.	Schell et al., 2017a
	Nikkomycin Z (completed clinical trial)	Inhibition of chitin synthase	*Blastomyces dermatitidis Coccidioides* sp.	Clemons and Stevens, 1997; Shubitz et al., 2014
GPI anchor biosynthesis	E-1210/APX001 (clinical trial)	Inhibition of inositol acyltransferase	*Candida* sp. *Aspergillus fumigatus Scedosporium prolificans Fusarium solani Paecilomyces lilacinus*	Miyazaki et al., 2011

*Drugs: *N*’-(3-bromo-4-hydroxybenzylidene)-2-methylbenzohydrazide, BHBM; 3-bromo-N0-(3-bromo-4-hydroxybenzylidene) benzohydrazide, D0; 4-{3-[1-(3-{4-[amino(imino)methyl]phenoxy}propyl)piperidin-4-yl]propoxy}benzamidine, T-2307. Clinical trials were accessed at the website https://clinicaltrials.gov.*

The majority of these molecules had been evaluated for their efficacy against fungi responsible for systemic and life-threatening diseases, such as *Candida* sp., *Cryptococcus* sp., and *Aspergillus fumigatus* (Pianalto and Alspaugh, 2016; McCarthy et al., 2017). However, few studies have evaluated the effect of these drugs in dermatophytes. In particular, VT-1161 is currently under phase I clinical trial for onychomycosis NCT02267356 (Garvey et al., 2015; Schell et al., 2017a). This drug inhibits the fungal cytochrome P-450 enzyme lanosterol 14-α-demethylase (Cyp51), being effective against several fungi including the dermatophytes *Trichophyton rubrum, Trichophyton mentagrophytes*, and *Epidermophyton floccosum* (Garvey et al., 2015). Moreover, VT-1161 has shown efficacy in a guinea pig infection model of dermatophytosis caused by *T. mentagrophytes* (Garvey et al., 2015). AR-12 constitutes an anti-tumor drug that inhibits acetyl coenzyme A (acetyl-CoA) synthetase, which is currently in clinical trial for lymphoma and solid tumors (NCT00978523). This drug also exhibits antifungal activity against several life-threatening fungi (**Table 1**) as well as the dermatophyte *T. rubrum* (Koselny et al., 2016; Kushwaha et al., 2017). In addition, AR-12 is able to reach the human nail plate and is thus considered a promising drug to treat onychomycosis (Kushwaha et al., 2017).

Moreover, plant kingdom diversity is widely unexplored and can serve as a source for almost limitless compounds. In particular, many plant secondary metabolites have drawn attention as being endowed with antifungal properties. In addition, these natural sources lead to novel structural entities, which can be used directly or function as a precursor for the development of better and more effective molecules (Arif et al., 2009; Negri et al., 2014). The identification of promising molecules with antifungal properties can be streamlined through the screening of crude plant extract to aid the discovery process as the first step of investigation, followed by assays geared toward the identification of molecules responsible for the antifungal activity (Liu et al., 2009; Medeiros et al., 2011; Negri et al., 2014). Notably, this process has provided some evidence of the biological properties of plant extracts and their phytochemical compounds against dermatophytes (**Table 2**).

**Table 2:** Antifungal compounds derived from plants extracts showing activity against dermatophytes.

Plant	Phytochemical	Dermatophyte	MIC values	Reference
*Thymbra capitata*	EO, carvacrol (>65%)	5 dermatophyte strains	0.08–0.32 μL/mL	Salgueiro et al., 2004
*Juniperus* sp.	EO, α-pinene (>48%)	*E. floccosum*	0.08–2.5 μL/mL	Cavaleiro et al., 2006
		*Trichophyton* sp.		
		*Microsporum* sp.		
*Lippia gracilis* LGRA-106	EO, thymol (>61%)	*T. rubrum*	23.44–46.87 μL/mL	de Melo et al., 2013
*Lippia gracilis* LGRA-109	EO, carvacrol (>54%)	*T. rubrum*	46.87–93.75 μL/mL	de Melo et al., 2013
*Thymus vulgaris*	EO thymol (>48%)	*Trichophyton* sp.	NT^∗^	Sokovic et al., 2008
*Vernonanthura tweedieana*	sesquiterpene (6-cinnamoyloxy-1-hydroxyeudesm-4-en-3-one)	*T. mentagrophytes* *M. gypseum*	4–8 μg/mL	Portillo et al., 2005
*Zuccagnia punctata*	Chalcones	*Trichophyton* sp.	8–16 μg/mL	Svetaz et al., 2007
		*M. gypseum*		
*Glaucium oxylobum*	Alkaloids	*Trichophyton* sp.	300 μg/mL	Morteza-Semnani et al., 2003
		*Microsporum* sp.		
		*E. floccosum*		
*Tabernaemontana catharinensis*	MMV alkaloid	*T. rubrum*	0.16 mg/mL	Medeiros et al., 2011
*Baccharis darwinii*	Diversinin (coumarin)	*Trichophyton* sp.	15.6 μg/mL	Kurdelas et al., 2010
		*M. gypseum*		
*Baccharis darwinii*	Anisocoumarin H	*Trichophyton* sp.	62.5 μg/mL	Kurdelas et al., 2010
		*M. gypseum*		
*Lavandula viridis L’He′r*	Monoterpenes	*Trichophyton* sp.	0.32–0.64 μL/mL	Zuzarte et al., 2011
		*Microsporum* sp.		
		*E. floccosum*		
*Piper ecuadorense*	Pinocembrin	*Trichophyton* sp.	125 μg/mL	Ramirez et al., 2013
*Dorstenia barteri*	Isobavachalcone	*T. rubrum*	1.2 μg/mL	Mbaveng et al., 2008
		*M. audorium*		
*Dorstenia barteri*	Amentoflavone	*T. rubrum*	39.1 μg/mL	Mbaveng et al., 2008
*Terminalia chebula*	Apigenin	*T. mentagrophytes*	NT^∗^	Singh et al., 2014
*Piper solmsianum*	Orientin	*Microsporum* sp.	≤9 μg/mL)	De Campos et al., 2005
		*Trichophyton* sp.		
		*E. floccosum*		

*^∗^NT, MIC values non-tested, but antifungal activity evaluated through *in vivo* assays.*

In particular, plant metabolites possess diverse anti-infective activity. Coumarins exhibit immunoregulatory effects toward macrophages, whereas quinones bind with adhesins and cell wall polypeptides, impairing their function. Saponines are grouped into three main categories: triterpenoid, steroid, or steroidal glycoalkaloid, and act by disrupting sterol-containing membranes, leading to a breakdown and loss of cellular integrity (Keukens et al., 1995; Arif et al., 2009). Moreover, phenolic compounds exert activity on different biological pathways and cellular targets, as previously reported against *Candida* sp. For example, cinnamic acid has an immunoregulatory effect on the activation of monocytes (Conti et al., 2013), curcumin and isoquercitrin cause cellular membrane damage (Lee and Lee, 2014; Yun et al., 2015), licochalcone A leads to the impairment of hyphae development (Teodoro et al., 2015), and caffeic acid inhibits isocitrate lyase (Cheah et al., 2014). In addition, the blocking of drug transporters pumps by curcumin (Sharma et al., 2009), thymol, carvacrol, and baicalein has also been reported (Teodoro et al., 2015).

The phytochemical effects on different cellular targets have also prompted investigation of the actions of these compounds toward dermatophytes. Studies have demonstrated fungitoxicity of the monoterpene linalool toward *T. rubrum* (de Oliveira Lima et al., 2017), as well as that of thymol and carvacrol; moreover, the essential oils from two *Lippia gracilis* genotypes (LGRA-106 and LGRA-109) presented antidermatophytic activity similar to that of FLC (de Melo et al., 2013). In addition, the MMV alkaloid (12-methoxy-4-methylvoachalotine) showed antifungal activity against *T. rubrum* (Medeiros et al., 2011). Finally, the chalcones, which comprise open-chain flavonoids, exhibit antifungal activity toward dermatophytes through the inhibition of cell wall biosynthesis (Boeck et al., 2005), and the blockage of FAS (Bitencourt et al., 2013).

Other studies have also attempted to assess the detailed modes of action of biomolecules against *T. rubrum*. For example, the glycoalkaloid α solanine showed antifungal activity toward *T. rubrum*, causing the down-regulation of the *erg1, erg11, mep4*, and *mdr2* genes, which were evaluated in a co-culture of *T. rubrum* with a keratinocyte cell line (Komoto et al., 2015). Caffeic acid also demonstrated antifungal activity against *T. rubrum*, with its mode of action appearing to be related to a decrease in ergosterol content, impairing the cellular membrane, along with a moderate inhibition of isocitrate lyase activity (Cantelli et al., 2017). Similarly, the flavonoids quercetin and luteolin showed activity against *T. rubrum* as well. Quercetin was assayed more comprehensively; the data hinted that its mode of action was related to ergosterol reduction, membrane damage, and the inhibition of FAS activity (Bitencourt et al., 2013). In this context, a *trans*-chalcone compound also simultaneously inhibited the synthesis of fatty acid and ergosterol, and showed marked inhibitory activity against strains of *T. rubrum* (Bitencourt et al., 2013; Komoto et al., 2015). Moreover, licochalcone A presented marked antifungal activity toward *T. rubrum*, causing the down-regulation of genes encoding putative virulence factors such as isocitrate lyase, citrate synthase, and malate synthase as well as of genes involved in cell wall synthesis and ergosterol biosynthesis. In addition, this compound impaired hyphal development during the interaction of *T. rubrum* with a keratinocyte cell line, and markedly inhibited the isocitrate lyase activity (Cantelli et al., 2017). Finally, chalcone treatment led to a down-regulation of a gene coding for a drug efflux pump during the co-culture of *T. rubrum* conidia with a keratinocyte cell line (Komoto et al., 2015).

Chalcones constitute widespread natural molecules that are a subject of increasing interest owing to their numerous pharmacological activities (Nowakowska, 2007). These compounds are considered interesting and promising molecules, and their simple chemical structures allow their synthesis in a secure, inexpensive, and straightforward manner (Narender and Reddy, 2007). They exhibit antifungal activity against both different and specific fungal targets, and also play a role as MDR modulators. For example, the inhibition of an MDR efflux pump (P-glycoprotein – P-gp) by chalcones was previously assessed through testing distinct methoxy-chalcones B-ring derivatives in a human *mdr1* gene transfected into a mouse lymphoma cell line, which showed a high binding affinity to P-gp, exceeding that of a known MDR blocking agent (Ivanova et al., 2008). Moreover, the use of chalcone analogs also promoted the sensitization of a fluconazole-resistant *C. albicans* strain mediated by the inhibition of efflux pumps (Lacka et al., 2015; Wang et al., 2016).

Taking into account the features of natural compounds together with the increasing need for new drugs, which has been mainly forestalled owing to the challenges afforded by similarities shared between mammalian and fungal cells, these molecules, and their derivatives appear as promising alternative sources for the development of new antifungal agents. Additionally, their combinatory effects with synthetic and the commercial drugs may represent new strategies for antifungal therapies.

## Mechanisms of Resistance

### Drug Exposure, Stress Responses, and Adaptation

Fungi must sense and respond appropriately to environmental changes to survive cellular stresses, such as those caused by drug exposure. They have evolved diverse mechanisms for adaptation with complex circuitries involving cellular responses, including the interplay among signaling molecules, stress responses, and drug resistance (Cowen, 2008). Following drug exposure, cell wall instability, changes in osmolarity, and production of reactive oxygen species are among the stimuli that can activate signaling pathways, such as the cell wall integrity pathway, calcineurin signaling, and high osmolarity glycerol (HOG) pathway to counteract the effects of drug-induced stress (Hayes et al., 2014). Critical signaling pathways involved in the tolerance to antifungal agents are modulated by stress responses governed by the molecular chaperone Hsp90, a global cellular regulator that controls the stability and activation of key regulators of stress responses, such as calcineurin and the MAP kinase in the Pkc1 cell wall integrity pathway, Mkc1 (Robbins et al., 2017).

Among pathogenic fungi, most of the known molecular mechanisms underlying drug stress response, drug tolerance, or resistance were initially elucidated in *Candida* sp., *Aspergillus* sp., and *Cryptococcus* sp., and they are still poorly understood in dermatophytes. In particular, it is still challenging to utilize gene targeting to study gene function in most species of this group of fungi; thus, transcriptomics have been applied to better understand the molecular mechanisms involved in drug-stress responses and adaptation. For example, *T. interdigitale* and *T. rubrum* respond to sub-lethal doses of several antifungal compounds by modulating the expression of genes related to various processes, such as protein transport, drug efflux, lipid metabolism, signal transduction, translation, post-translational modifications, and oxidative stress, depending on the drug and time of exposure (Paião et al., 2007; Yu et al., 2007a; Zhang et al., 2007, 2009; Peres et al., 2010b; Persinoti et al., 2014; Mendes et al., 2018). Specifically, a gene encoding a transposable element was shown to be up-regulated in *T. interdigitale* after exposure to several drugs (Paião et al., 2007; Peres et al., 2010b). In turn, transposable elements can influence gene expression; in *Schizosaccharomyces pombe*, the preferential integration of the retrotransposon Tf1 into promoters of stress response genes has been reported, thereby enhancing their expression (Feng et al., 2013). However, the regulatory mechanisms involved in stress responses are complex. Recently, a refined use of pre-mRNA processing events has been reported in *T. rubrum* in response to undecanoic acid (UDA), regulating the expression of genes encoding phosphoglucomutase, which is involved in gluconeogenesis and in cell wall biosynthesis, along with inosine monophosphate dehydrogenase, a key enzyme supplying guanine nucleotides to cells (Mendes et al., 2018). These mechanisms, along with others, collectively help the cells to counteract the toxicity of drugs and cope with stress.

It has been reported that the repeated exposure of *T. rubrum* to sub-lethal doses of antifungal drugs *in vitro* can lead to the selection of strains able to survive higher minimal inhibitory concentrations (MICs) following drug pressure. However, in the absence of such pressure, a fraction of the strains regained susceptibility (Ghelardi et al., 2014). The strains that survive higher MICs can be classified either as tolerant, being able to survive transient exposure, or resistant, having acquired mutations that confer the stable ability to survive higher MICs (Delarze and Sanglard, 2015). In particular, in *Candida* sp. and *Aspergillus* sp., Hsp90 promotes drug tolerance by stabilizing key regulators of cellular stress responses (Robbins et al., 2017), thus controlling the downstream signaling pathways.

Therefore, fungal survival in inhospitable environments, as in the presence of antifungal agents, relies on the stress responses and involves a global modulation of gene expression, resulting in the synthesis of specific proteins that are fundamental to counteract stress. The relevance of these genes in the survival of fungi suggests that they may be considered as potential therapeutic targets. Here, we focus mainly on the drug stress response related to drug efflux, drug detoxification, and the modulation of kinase and heat shock (*hsp)* genes.

### Drug Efflux

Efflux cell membrane transporters are proteins capable of binding to different compounds including antifungal drugs, and subsequently extruding them from the cell. The expression of these molecular pumps represents one of the main reasons for treatment failure, acting as a protective mechanism against the cytotoxic effects of the drug by reducing the accumulation of harmful compounds (Putman et al., 2000; Cervelatti et al., 2006; El-Awady et al., 2016). The efflux cell activity leads to a gradual and non-specific increase in drug resistance by diminishing the expected effect *in vivo*.

In turn, enhanced drug export represents a critical strategy among the mechanisms used by fungi to gain resistance toward antifungal therapy (Sharma and Prasad, 2011). The modification of efflux transporters in fungal resistance contributes to the pathogenicity, favoring the occurrence of cases of dermatophytosis by providing a colonization advantage to the fungus (Coleman and Mylonakis, 2009). In particular, MDR results to a large degree from the overexpression of genes belonging to the ATP-binding cassette superfamily (ABC), which is evolutionarily conserved in both prokaryotes and eukaryotes. These proteins contain two distinct regions: a highly conserved nucleotide-binding domain and a quite variable transmembrane domain. Through the binding and hydrolization of ATP, ABC transporters actively move a wide variety of structurally and chemically unrelated compounds across membranes, thereby reducing drug accumulation even in cancer cells (Martinez-Rossi et al., 2008; Housman et al., 2014; Xiong et al., 2015). This superfamily encompasses three well-studied families involved in the efflux of toxic compounds: MDR, MDR-associated protein (MRP), and the pleiotropic drug resistance (PDR) families (Coleman and Mylonakis, 2009).

The availability of the genome sequence of different dermatophytes has revealed a homogeneous group of these transporters, which exhibits very low genetic diversity with only a few exclusive genes in each analyzed species (Burmester et al., 2011; Martinez et al., 2012). The analysis of seven of these dermatophyte genomes identified a large number of encoded ABC transporter domains with many of the associated genes having counterparts in all studied species, suggesting that ABC transporter genes act equivalently, despite the phenotypic and adaptive uniqueness of each species (Gadzalski et al., 2016).

Evidence implicating drug efflux as a mechanism of resistance has been extensively evaluated in the *T. interdigitale* H6 strain (previously identified as *T. rubrum*). Initially, the occurrence of a drug efflux phenomenon was hypothesized consequent to an observed resistance to both GRS and tioconazole *in vitro* (Fachin et al., 1996). Subsequently, *mdr1* and *mdr2* genes were identified, which presented an increased level of transcription after exposure to different classes of antifungal agents (Cervelatti et al., 2006; Fachin et al., 2006; Paião et al., 2007). The disruption of the *mdr2* gene rendered a mutant more sensitive to terbinafine (TRB) but not to other tested drugs, precluding the suggestion of a modulatory role of the *mdr2* gene in drug susceptibility (Fachin et al., 2006). Gene expression analysis later demonstrated that the *mdr4* gene somehow depends on the MDR2 transporter when challenged with antifungal agents, suggesting the existence of network interaction with regard to MDR activity as well as the interdependence of different ABC transporters in drug efflux (Yibmantasiri et al., 2014; Martins et al., 2016).

Different ABC transporters with overlapping substrate profiles may present broad ranges of substrate affinity and capacity of transport, and may respond differently to cellular drug-concentrations, thereby determining the actual fate of the substrates (Glavinas et al., 2004). For example, the transcriptional profiling of the *pdr1, mdr2*, and *mdr4* genes of four species of *Trichophyton*: *T. equinum, T. interdigitale, T. rubrum*, and *T. tonsurans*, did not reflect the intrinsic phylogenetic relationship among these fungi, nor was a functional correlation revealed between species and the efflux modulation under the tested conditions. However, the evaluated genes appear to work synergistically, leading to the understanding that one *mdr* gene may be compensated by others as related to extrusion activity (Martinez-Rossi et al., 2008; Gadzalski et al., 2016; Martins et al., 2016).

Owing to the relevant contribution of drug efflux resistance mechanisms to the pathogenesis of dermatophytes, the use of inhibitors or modulators of ABC transporters to block and reverse MDR response represents a potential alternative method to thwart multidrug transporter activity, thus preventing the occurrence of drug efflux (Glavinas et al., 2004; Sharma and Prasad, 2011; Holmes et al., 2016). An assortment of such compounds present MDR modulatory properties by synergistically enhancing fungicidal activity, although these still require a definitive demonstration of clinical efficacy. The identified inhibitors include isonitrile, enniatins, milbemycins, ibuprofen, and the calcineurin inhibitor FK506. These compounds present effective efflux blocking activity in a terbinafine-resistant strain of the dermatophyte *Microsporum canis* as well as in multiple types of eukaryotic cells including malignant cells (Kawahara et al., 2015; Holmes et al., 2016; Kano et al., 2018). However, it is relevant to consider that cells can heterogeneously express ABC transporters, varying the responses between fungal species, patients, as well as within tumors, potentially impairing their activity (Holmes et al., 2016; Martins et al., 2016). Moreover, the many similarities between fungal and human cells represent a challenge for host treatments in regard to the currently limited antifungal choices (Martinez-Rossi et al., 2008). As multidrug transporter genes are also relatively conserved from microorganisms to humans, an ideal MDR- interfering drug must therefore be capable of chemosensitizing cells to existing effective antifungal agents while also shielding human orthologs (Glavinas et al., 2004; Holmes et al., 2012). This approach represents a hopeful option for effective chemotherapy and a pivotal strategy to overwhelm clinical MDR outcomes.

### Drug Detoxification

A substantial group of genes involved in cellular detoxification is activated when fungi are challenged with cytotoxic drugs at sub-inhibitory concentrations, which contributes to increasing the tolerance to these drugs. RNA-sequencing (RNA-Seq) analysis of the effect of acriflavine (ACR) on *T. rubrum* showed that genes involved in cellular detoxification were highly up-regulated, including those encoding catalases that protect the cell against oxidative stress and reactive oxygen species (Persinoti et al., 2014). The increased expression of catalase genes may constitute a compensatory mechanism to maintain the intracellular level of this enzyme, thus protecting the cells from apoptotic effects elicited by the drug. Conversely, catalase activity is inhibited by acriflavine *in vitro*. Moreover, RNA-Seq analysis of the differential gene expression of *T. rubrum* challenged with UDA also revealed the up-regulation of several genes coding for antioxidant enzymes. In addition to catalases, superoxide dismutase, peroxidases, glutathione transferases, and glutathione peroxidases were identified as being stimulated in response to UDA, leading to enzymatic oxidative detoxification of the fungal cells (Mendes et al., 2018). Consistent with these findings, a previous report also suggested that *Candida* resistance to amphotericin B (AMB) might be mediated by increased catalase activity as well (Peman et al., 2009).

The ability of a fungus to secrete enzymes is related to its pathogenicity and stress response as well as its mechanisms of detoxification. *T. rubrum* secretes esterases and other enzymes depending on the nutrients supplied (Brasch et al., 1991). Furthermore, the electrophoretic pattern of esterases secreted from *T. rubrum* revealed new isoforms when this dermatophyte was grown in sub-inhibitory concentrations of GRS and tioconazole (Fachin et al., 2001). These additional forms of esterase may participate in the cellular detoxification directly or may comprise a non-specific cellular response to stress leading to drug tolerance. The participation of esterase in detoxification leading to drug resistance has already been described in other organisms (Harms et al., 1996; Rooker et al., 1996).

Overexpression of target enzymes is also found in several azole-resistant strains, which is a compensatory mechanism for ergosterol depletion. Overexpression of *erg11* occurs in *C. albicans* through duplication of the left arm of chromosome 5, which harbors the *erg11* gene (Cowen et al., 2015; Sanglard, 2016). *Cryptococcus neoformans* adapts to high concentrations of FLC by the formation of chromosome 1 disomies. This resistance is a consequence of the duplication of two genes that are located on this chromosome: ERG11, the target of FLC and AFR1, a transporter of azoles in *C. neoformans* (Sionov et al., 2010). Also, extra copies of the *A. fumigatus* squalene epoxidase gene confer resistance to terbinafine (Liu et al., 2004). However, extra copies of the salicylate 1-monooxygenase (*salA*) gene were suggested to be the cause of terbinafine resistance in *Aspergillus nidulans*, although this enzyme does not represent the target of this drug. Instead, the authors hypothesized that the cleavage of the naphthalene nucleus present in the molecular structure of terbinafine is responsible for its degradation and consequently the resistance phenotype (Graminha et al., 2004). Subsequently, a strain of *T. rubrum* was rendered resistant to terbinafine by transformation with multiple copies of the *salA* gene (Santos et al., 2018). This strain exhibited elevated expression of the *salA* gene and simultaneously increased resistance to terbinafine, compared to the original strain. Thus, these results showed that this gene plays a role in the resistance and response to terbinafine in dermatophytes. However, the resistant phenotype was reversed after the strain was cultured in the absence of terbinafine for several generations, thereby losing plasmids containing the *salA* gene, which confirmed that extra copies of this gene provoked resistance. Moreover, the original strain without any plasmid responded to the challenge with terbinafine by increasing the expression of the endogenous *salA* gene (Santos et al., 2018).

Unlike bacteria, which exhibit several examples of antibiotic resistance through the degradation of these compounds, the resistance of fungi to antifungal agents by inactivation or degradation is a not well known (Martinez-Rossi et al., 2008). Taking into account that fungi secrete a large number of enzymes (Martinez-Rossi et al., 2017), it is probable that similar mechanisms resulting in resistance or tolerance to antifungal may be frequent. The disclosure of fungal enzymes having this role may thus be useful for the development of enzyme inhibitors, which could be applied alone or in association with conventional therapy.

### Transcriptional Modulation of Kinase Genes

Genome-wide expression profiling has provided new targets for the development of novel drugs by the pharmaceutical industries and increased the general understanding regarding the mechanisms of action of different drugs and their therapeutic effects. Several studies have revealed the existence of various regulatory and metabolic circuits necessary to develop resistance to antifungal agents (Sanglard et al., 2009). Considering the nature of the pathogen stress-response systems and that some cellular proteins related to these mechanisms are phosphorylated, protein kinases may represent a potential target for antifungal drug therapy (Garaizar et al., 2006). In particular, protein phosphorylation is known to trigger several signaling transduction pathways involved in cell stimuli propagation. Thus, several groups have been exploring different genetic approaches to investigate the expression of kinases responsive to different drugs, with the purpose of obtaining a better understanding of dermatophyte resistance to antifungal agents.

For example, a microarray hybridization approach was applied in the analysis of gene expression responses of dermatophytes exposed to several antifungal agents. *T. rubrum* was exposed to sub-inhibitory concentrations of the antifungal agents ketoconazole and AMB (Yu et al., 2007b). The authors reported the down-regulation of four kinase genes in response to AMB, highlighting two genes coding for MAP kinases (MpkA and STE7). Notably, these proteins belong to different stress-responsive pathways. The authors also reported a down-regulated kinase gene in response to AMB, coding for a serine/threonine protein kinase (*sps1*). This protein is important for transcriptional, biochemical, and morphological events during the later events of fungal sporulation (Friesen et al., 1994; Widmann et al., 1999; Yu et al., 2007b). Conversely, *T. rubrum* cultivated in the presence of terbinafine showed an up-regulation of the kinase gene *sps1* (Zhang et al., 2007). These authors have also reported a significant down-regulation of two-phosphorylated-intermediary-components involved in regulating a MAP kinase cascade, as well as a predicted unusual protein kinase (Yu et al., 2007b; Zhang et al., 2007).

In addition, the same group analyzed the transcription of *T. rubrum* genes in response to PHS11A and PH11B, inhibitors of synthesized fatty acid synthase (FAS), which was chosen as an interesting antifungal target owing to architectural differences between the mammalian and fungal protein. Four kinase genes were negatively modulated in response to PHS11A: *pkaC, ssp1*, and *sln1*, which are related to signal transduction pathways, and a putative dihydroxyacetone kinase (DAK2), related to carbohydrate transport and metabolism. Only one kinase gene (*bck1*) showed up-regulation in the presence of PHS11A. *Bck1* was also responsive to TRB (Zhang et al., 2007, 2009). In turn, PH11B up-regulated two kinase genes, the pyrophosphate-dependent phosphofructo-1-kinase gene (*pfk1*) and a hexokinase gene (*glk1*). Both genes are related to carbohydrate transport and metabolism (Yu et al., 2007a).

Up-regulation of the MAP kinase gene (*mpka*) in the presence of itraconazole has also been reported (Diao et al., 2009). This gene was also down-regulated in response to AMB, being related to cell wall integrity. Thus, the authors argued that the stress caused by this antifungal agent in the ergosterol pathway, and consequently in the plasma membrane, can be compensated by the activation of the cell wall integrity pathway, altering the structure and composition of the fungal cell wall (Jung and Levin, 1999; Yu et al., 2007b; Diao et al., 2009).

Moreover, a suppressive subtractive hybridization approach was used to identify genes differentially expressed after the exposure of *T. rubrum* to the cytotoxic drugs ACR, fluconazole (FLC), GRS, TRB, and UDA (Paião et al., 2007). The authors described the up-regulation of the never in mitosis A (*nima*) gene in response to FLC, GRS, and TRB. This gene was also up-regulated in *T. rubrum* under acidic pH and growth in keratin as the only carbon source (Paião et al., 2007; Peres et al., 2010b). The NIMA kinase family, initially isolated from *A. nidulans*, is required for the G2/M transition. Mammalian NIMA-related kinase 11, long and short isoform (*nek11L* and *nek11S*) genes are related to DNA replication/damage stress response, being also expressed in response to unspecific cellular stresses (Osmani et al., 1988; Noguchi et al., 2002; Paião et al., 2007; Peres et al., 2010b).

Differential display RT-PCR and northern blot techniques have been used to analyze *T. rubrum* molecular responses to acriflavine as well, showing increased transcription levels of a gene coding for a putative riboflavin kinase. This enzyme is essential in the flavin biosynthetic pathway, catalyzing the first step of this process yielding flavin mononucleotide (FMN) and flavin adenine dinucleotide (FAD). This pathway is crucial for several biological processes including lipid, protein, and carbohydrate metabolism (Santos et al., 2000; Segato et al., 2008).

In turn, a high-throughput RNA-Seq approach was applied to evaluate the time-dependent effects of acriflavine on the *T. rubrum* transcriptome. In 3 h of drug exposure, four kinase genes responded to acriflavine, two being up- (*adenosine 5′-phosphosulfate kinase* and *homoserine kinase*) and two down-regulated (protein kinase subdomain-containing protein, and pyridoxine kinase). In 12 h of exposure, only one kinase gene was up-regulated (*cmgc/srpk protein kinase*). In 24 h of exposure, four kinase genes were up-regulated, highlighting the genes coding for a calcium-calmodulin-dependent pathway, indicating that acriflavine probably affects this signaling route in *T. rubrum*, which is reported to be involved in cell survival under stressful conditions (Persinoti et al., 2014). Moreover, in 24 h of exposure, five kinase genes were down-regulated. Notably, acriflavine repressed an unusual kinase gene (coding for an atypical/ABC1/ABC1-B protein kinase) that does not display most of the typical eukaryotic protein kinase features, being reported as a chloroplast or mitochondria-related protein that regulates quinone synthesis (Huang et al., 2015). In addition, a gene coding for a kinase subdomain-containing protein was strongly up-regulated upon acriflavine exposure (Persinoti et al., 2014).

Recently, RNA-Seq analysis also revealed interesting data related to the response of *T. rubrum* to sub-lethal doses of UDA (Mendes et al., 2018). In this study, three kinase genes were up-regulated including the uridine/cytidine kinase (*uck*), *ste/ste7* (*ste7*) protein kinase, and dephospho-CoA kinase (*dpck*). UCK is involved in the conversion of both cytidine and uridine to nucleoside monophosphate. STE7 is associated with the MAPK pathway, and DPCK is involved with the final steps of the pathway for CoA biosynthesis (Wadler and Cronan, 2007; Tomoike et al., 2017; Mendes et al., 2018). One gene was down-regulated (nucleoside diphosphate kinase) in the early response to UDA (3 h). At 12 h of exposure to UDA, two distinct serine/threonine protein kinase coding genes were down-regulated. Furthermore, the authors reported two kinase genes responding to UDA in both 3 and 12 h of drug exposure; one was another distinct serine/threonine protein kinase coding gene, showing a strong down-regulation. The other was AGC/RSK protein kinase, which was also down-regulated in response to acriflavine (Persinoti et al., 2014; Mendes et al., 2018). AGC protein kinase (a subgroup of the Ser/Thr protein kinases) was named based on sequence alignments of the catalytic domain with the cAMP-dependent protein kinase 1 (PKA), cGMP-dependent protein kinase (PKG), and protein kinase C. Notably, ribosomal S6 kinase (RSK), a member of the AGC family, contains two kinase catalytic domains in the same polypeptide. Thus, in addition to the AGC kinase domain at the N-terminal portion, the C-terminus displays a calcium/calmodulin dependent kinase domain that is activated by the extracellular signal-regulated kinase (eRK) pathway and plays an important role in protein synthesis (Pearce et al., 2010). All of these kinase genes/proteins described above are listed in **Table 3**.

**Table 3:** Relation of *T. rubrum* kinase genes modulated by antifungal agents.

Gene product name	ID	Antifungals	Modulation	Reference
Mitogen-activated protein kinase (MpkA)	TERG_00832	AMB, ITRA	Down-regulated, Up-regulated	Yu et al., 2007b, Diao et al., 2009
Mitogen-activated protein kinase (STE7)	TERG_02515	AMB	Down-regulated	Yu et al., 2007b
Serine/threonine protein kinase (SPS1)	TERG_00281	AMB	Down-regulated	Yu et al., 2007b
cAMP-dependent protein kinase catalytic subunit (PKaC)	TERG_03685	PHS11A	Down-regulated	Zhang et al., 2007
Suppressor of sensor kinase (SLN1)	TERG_06080	PHS11A	Down-regulated	Zhang et al., 2007
Serine/threonine protein kinase (SSP1)	TERG_05979	PHS11A	Down-regulated	Zhang et al., 2007
Dihydroxyacetone kinase (DAK2)	TERG_08455	PHS11A	Down-regulated	Zhang et al., 2007
Mitogen-activated protein kinase (BCK1)	TERG_00207	PHS11A	Up-regulated	Zhang et al., 2007
Pyrophosphate-dependent phosphofructo-1-kinase (PFK1)	TERG_05437	PH11B	Up-regulated	Yu et al., 2007a
Hexokinase (GLK1)	TERG_08933	PH11B	Up-regulated	Yu et al., 2007a
Never in mitosis A kinase (NIMA)	TERG_06149	FLC, GRS or TRB	Up-regulated	Paião et al., 2007
Riboflavin kinase (FMN1)	TERG_03281	ACR	Up-regulated	Segato et al., 2008
Pyridoxine kinase	TERG_06151	ACR	Down-regulated	Persinoti et al., 2014
Adenosine 5’-phosphosulfate kinase	TERG_07921	ACR	Up-regulated	Persinoti et al., 2014
Kinase subdomain-containing protein	TERG_07159	ACR	Down-regulated	Persinoti et al., 2014
Kinase subdomain-containing protein	TERG_02787	ACR	Up-regulated	Persinoti et al., 2014
Kinase subdomain-containing protein	TERG_01497	ACR	Up-regulated	Persinoti et al., 2014
Mitogen-activated protein kinase (MAF1)	TERG_00595	ACR	Up-regulated	Persinoti et al., 2014
CMGC/SRPK protein kinase	TERG_08979	ACR	Up-regulated	Persinoti et al., 2014
Calcium-calmodulin-dependent protein kinase (CamK)	TERG_02198	ACR	Up-regulated	Persinoti et al., 2014
CAMKK protein kinase (CamKK)	TERG_07555	ACR	Up-regulated	Persinoti et al., 2014
Stress activated MAP kinase interacting protein	TERG_03684	ACR	Down-regulated	Persinoti et al., 2014
Atypical/ABC1/ABC1-B protein kinase	TERG_07639	ACR	Down-regulated	Persinoti et al., 2014
Serine/threonine protein kinase	TERG_04042	ACR	Up-regulated	Persinoti et al., 2014
Serine/threonine protein kinase	TERG_07140	ACR	Down-regulated	Persinoti et al., 2014
Thiamine pyrophosphokinase	TERG_05058	ACR	Down-regulated	Persinoti et al., 2014
Uridine/cytidine kinase (UCK)	TERG_05622	UDA	Up-regulated	Mendes et al., 2018
STE/STE7 protein kinase (STE7)	TERG_08066	UDA	Up-regulated	Mendes et al., 2018
Dephospho-CoA kinase (DPCK*)*	TERG_04392	UDA	Up-regulated	Mendes et al., 2018
Nucleoside diphosphate kinase (NDK)	TERG_04558	UDA	Down-regulated	Mendes et al., 2018
Serine/threonine protein kinase	TERG_08278	UDA/ACR	Down-regulated	Persinoti et al., 2014; Mendes et al., 2018
Serine/threonine protein kinase	TERG_00199	UDA	Down-regulated	Mendes et al., 2018
Serine/threonine protein kinase	TERG_03851	UDA	Down-regulated	Mendes et al., 2018
AGC/RSK protein kinase	TERG_00281	UDA/ACR	Down-regulated	Persinoti et al., 2014; Mendes et al., 2018

*Drugs: ACR, acriflavine; AMB, amphotericin B; FLC, fluconazole; GRS, griseofulvin; ITRA, itraconazole; TRB, terbinafine; UDA, undecanoic acid, and PHS11A and PH11B.*

### Fungal Heat Shock Proteins

Initially discovered in *Drosophila* (De Maio et al., 2012), the heat shock proteins (Hsps) comprise chaperones present in all organisms studied to date that participate in diverse cellular processes such as transcription, translation, post-translational modifications, protein folding, and protein aggregation and disaggregation (Tiwari et al., 2015). Some Hsps may be induced when cells undergo stressful conditions, such as the exposure to antifungal agents, and extremes of temperature or pH (Singh et al., 2009; Leach et al., 2012a; Tamayo et al., 2013; Tiwari et al., 2015). These proteins are classified according to their functions and molecular mass into six families: Hsp40, Hsp60, Hsp70, Hsp90, Hsp100, and small Hsps (Tiwari et al., 2015). In fungi, Hsp20-40, Hsp70, and Hsp90 are predominant and participate in several cellular events, with some Hsps acting as co-chaperones (Fink, 1999; Tiwari et al., 2015). In *C. albicans*, low molecular mass Hsps are ATP independent, whereas those with higher molecular mass are ATP dependent (Gong et al., 2017). Usually, the *hsp* genes are activated by a heat shock transcription factor (HSF). This protein under stress conditions is phosphorylated and trimerized, binding to the promoter regions of the *hsp* genes and inducing their transcription (Akerfelt et al., 2010).

The relationship between the gene expression of *hsp* genes and the stress induced by antifungal drugs has been evidenced in different pathogenic agents including yeasts and filamentous fungi (Tiwari et al., 2015). In dermatophytes, the exposure to sub-lethal doses of diverse cytotoxic drugs results in the modulation of several *hsp* genes (Martinez-Rossi et al., 2016). Drug stress induces the up-regulation of genes encoding proteins belonging mainly to the Hsp70 family, such as in response to TRB, PHS11A, and ACR, or belonging to the small Hsp family, such as in response to TRB, AMB, ITR, and ACR (Jacob et al., 2015; Martinez-Rossi et al., 2016). Hsp70 proteins are monomeric and collaborate with the Hsp40 family of co-chaperones to bind to linear segments of unfolded proteins, whereas small Hsps form oligomeric complexes that bind to denatured proteins (Schopf et al., 2017). The genes encoding Hsp90 (Jacob et al., 2015), Cdc37, and the transcription factor Hsf1 (Martinez-Rossi et al., 2016) are up-regulated in *T. rubrum* in response to TRB and ACR. Cdc37 is one of the co-chaperones that regulates Hsp90 function, playing roles in the maturation of kinases (Schopf et al., 2017). Additionally, the genes encoding the transcriptional regulator Hsf1 and PacC are upregulated when *T. rubrum* and *T. interdigitale* are submitted to temperature shifts in keratin cultures. In a pacC^-^ strain of *T. interdigitale* the transcription of the *hsf1* gene was tightly reduced compared to the other *hsp* genes. The authors suggested that the collaboration between PacC and Hsf1 proteins modulates the level of *hsp* transcripts in response to cellular stress (Martinez-Rossi et al., 2016). In contrast, *T. rubrum* challenged with sub-inhibitory concentrations of UDA for 3 and 12 h did not significantly increase the expression of any *hsp* gene (Mendes et al., 2018), indicating that the cell used another strategy to cope with the stress provoked by this drug. In support of this, other pathways were also highlighted during UDA detoxification in *T. rubrum* such as those involved in lipid metabolism, cellular membrane composition, pathogenesis, and oxidative stress. Moreover, events of alternative splicing, including intron retention and exon skipping, were described in response to UDA as well (Mendes et al., 2018).

Hsp90, one of the most conserved Hsps, associates with a wide range of client proteins, controlling their activities by folding them to their active conformations. Clients are enriched in signal transducers including kinases and transcription factors (Taipale et al., 2010; Leach et al., 2012a). In *S. cerevisiae*, Hsp90 interacts with approximately 10% of the yeast proteome (Zhao et al., 2005; Leach et al., 2012b). By controlling the activation of key molecules, Hsp90 plays roles in diverse processes including morphogenetic changes, stress adaptation, and antifungal resistance (Cowen, 2009; Singh et al., 2009; Lamoth et al., 2016). In yeast, it was established that Hsp90 potentiates azole resistance in *C. albicans* and *Saccharomyces cerevisiae* (Cowen and Lindquist, 2005; Cowen, 2009), in addition to governing the cellular circuits required for resistance to echinocandins in *C. albicans* (Singh et al., 2009). This biological response occurs through the activation of calcineurin phosphatase by Hsp90 under stress conditions (Cowen, 2008; Singh et al., 2009). In *A. fumigatus*, the genetic repression of Hsp90 showed that this chaperone appears to be essential for the development of this fungus, causing a decrease in spore viability, reduction in hyphae growth, and defects in germination (Lamoth et al., 2012). Some information about the cellular location of Hsp90 in different pathogenic fungi may help to understand the biological functionality of this protein against toxic agents. In *C. neoformans*, Hsp90 is located on the cell surface, so that the inhibition of this protein confers the natural resistance to cell wall inhibitors (Chatterjee and Tatu, 2017). However, in *A. fumigatus*, Hsp90 is predominantly found in the cytosol, although in the presence of cell wall stressors it accumulates in the cell wall and the septa regions of the hypha, indicating an activity in compensatory stress mechanisms (Lamoth et al., 2012).

However, despite the well-established data regarding Hsp90 in drug-induced responses in several fungi, few studies have addressed protein interactions and their biological role in dermatophytes. For example, the chemical inhibition of Hsp90 of *T. rubrum* caused increased susceptibility to micafungin and itraconazole, and improved the efficacy of these antifungal agents. Additionally, Hsp90 inhibition impaired fungal growth in human nail fragments and altered the modulation of other *hsp* genes as well as the *pacC* gene, which encodes a transcription factor involved in fungal pathogenicity (Bignell et al., 2005; Ferreira-Nozawa et al., 2006), suggesting that Hsp90 is involved in both pathogenicity and drug susceptibility in *T. rubrum* (Jacob et al., 2015). In this case, the synergism observed between the inhibition of Hsp90 and the increased susceptibility to some antifungal in *T. rubrum* revealed Hsp90 as a novel target and its inhibition as a potential strategy to treat dermatophytosis (Jacob et al., 2015).

Although they present a high degree of homology, the differences between Hsp90 orthologs may be explained owing to the occurrence of post-translational modifications that can interfere in the differential regulation of these proteins (Lamoth et al., 2016). Considering the participation of Hsp90 in the development of drug resistance, drugs targeting this chaperone have been recognized as new therapeutic alternatives against pathogenic fungi. Currently, inhibitors and anti-Hsp90 antibodies have been shown to interfere with the activity of Hsp90 (Gong et al., 2017) by binding to the N-terminal region in the ATP binding site (Li et al., 2015); these agents include radicicol (RAD), geldanamycin (GdA), GdA analogs, and non-GdA Hsp90 inhibitors (Gong et al., 2017). However, for pharmacological application in clinical therapy, the toxicity of these drugs must be carefully evaluated in humans (Uehara, 2003) to avoid any potential for harm to patient health.

### Mutations Affecting Drug Target Genes

Several mutations in genes encoding enzymes targeted by antifungal agents, mostly enzymes involved in ergosterol biosynthesis and cell wall synthesis, have been described in different fungal pathogens. Azoles inhibits lanosterol alfa-14-demethylase (Cyp51) by binding the nitrogen in the azoles ring to the iron atom of the heme group of the protein. This class of drugs is used for the treatment of several mycoses, including dermatophytosis, owing to its broad spectrum of activity; however, an increasing number of resistant isolates have arisen (Campoy and Adrio, 2017). Mutations in the *erg11* gene, which encodes the lanosterol alfa-14-demethylase, are often found in *C. albicans* azole-resistant clinical isolates, resulting in non-synonymous amino acid substitutions in regions close to the active site of the enzyme. Although inducing post-translational modifications that affect its three-dimensional structure (Morio et al., 2010), these mutations do not affect the enzyme function and are often followed by loss of heterozygosity, which increases azole resistance (Selmecki et al., 2010). Moreover, *erg11* mutations have different effects on the affinity of distinct azole drugs, which interact with specific Erg11 residues (Morio et al., 2010). Accordingly, a recent study demonstrated that some mutations alter the hydrogen bond between the drug and target protein, reducing the affinity of short-tailed but not of long-tailed triazoles (Sagatova et al., 2016).

Another target enzyme of antifungal agents is squalene epoxidase (SE), which participates in the ergosterol biosynthesis pathway by catalyzing the epoxidation of squalene to 2-3-oxidosqualene. Terbinafine, the drug of choice for dermatophytes, is an antifungal agent that inhibits SE fungal activity in a non-competitive way, leading to depletion of ergosterol and accumulation of squalene (Favre and Ryder, 1996). Mutations in the gene for SE that give rise to amino acid substitutions lead to structural changes and decreased binding of terbinafine to the protein without causing dysfunction in ergosterol biosynthesis (Martinez-Rossi et al., 2008). Until recently, few *T. rubrum* clinical isolates resistant to terbinafine have been described, and only two clinical isolates were investigated and associated with SE mutations, which resulted in the amino acid substitutions L393F and F397L. Both isolates were cross-resistant to all tested SE inhibitors, with these mutations being in the same region as several mutations identified previously in terbinafine-resistant *S. cerevisiae* strains. In addition, the expression of *C. albicans* SE harboring mutations with corresponding amino acid substitutions was shown to decrease inhibitor susceptibility in *S. cerevisiae*; furthermore, single point mutations in *A. nidulan*s and *A. fumigatus* in amino acids corresponding to the F397L substitution were associated with terbinafine resistance (Osborne et al., 2005; Rocha et al., 2006; Martinez-Rossi et al., 2008) These data therefore suggested that this region may contain the high-affinity site for TRB.

**FIGURE 1:**
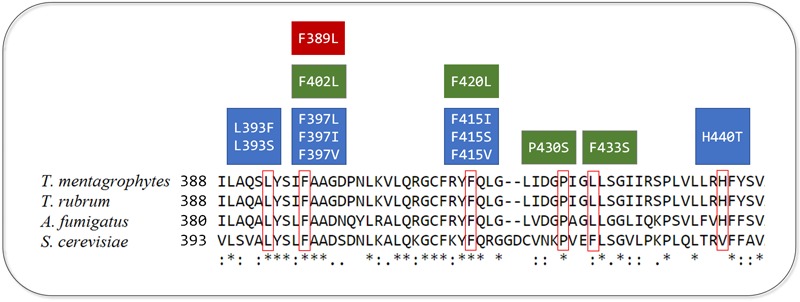
Alignment of amino acid sequences of squalene epoxidase (SE) of *T. mentagrophytes, T. rubrum, A. fumigatus*, and *S. cerevisiae*. The six residues with point mutations associated with terbinafine resistance are indicated by red contour. The boxes represent point mutations in SE from *T. mentagrophytes* and *T. rubrum* (blue), *A. fumigatus* (red), and *S. cerevisiae* (green). Asterisks are related to identical residues in the four species, whereas two dots or one dot represent a higher and lower degree of conservation, respectively.

Recently, 2,056 isolates of *T. rubrum* and *T. interdigitale* were evaluated regarding to their susceptibility to TRB. The authors found that only 17 isolates, representing less than 1%, were resistant to TRB. By analyzing the gene sequence of the SE, they found point mutations in four positions responsible for the resistant phenotype, which are represented in **Figure 1**. In addition to L393F and F397L, seven new mutations were identified including one in residue L393 and two in residue F397. These mutations were investigated through expression of the corresponding amino acid substitutions using *Arthoderma vanbreuseghemii* (*T. mentagrophytes*) as a recipient organism. The strains harboring mutated genes were less susceptible to TRB, with global gene expression analysis revealing no other significant differences between the mutated and control strains, indicating that the increased TRB resistance was due to the mutations (Yamada et al., 2017).

The substitution in the amino acid corresponding to F415 in *S. cerevisiae* (F420) has been previously associated with TRB resistance (Martinez-Rossi et al., 2008), being the first mutation found in residue His440. A valine is located in the equivalent position (**Figure 1**), which was not identified in a previous screening of critical amino acids by 3D modeling of *S. cerevisiae* SE (Nowosielski et al., 2011), owing to subtle conformational differences between the enzymes from *S. cerevisiae* and *Trichophyton* spp. Moreover, the high frequency of TRB resistant isolates in this study are considered to be related to the prolonged exposure during treatment, as eight among 17 patients had been previously treated with TRB (Yamada et al., 2017).

Currently, the increasing resistance to azoles in some *Candida* species has rendered echinocandins as the first line therapy for invasive candidiasis. This class of drugs inhibits the fks subunit of glucan synthases; accordingly, clinical resistance has been associated with mutations in fks-encoding genes. In *C. glabrata*, the majority of mutations are located in one of the two hot spot regions of *fks*1 and *fks*2, whereas in other *Candida* spp., these substitutions are encountered only in *fks*1, in two hot spot regions comprising residues Phe641–Pro649 and Arg1361. The most abundant mutations that give rise to the most pronounced phenotypes in *C. albicans* are substitutions in Ser645 and Phe641 (Perlin, 2015). This mechanism of resistance to echinocandins has also been described for *A. fumigatus*, through S678P substitution in the Fks1 subunit. In addition, the reduced intrinsic susceptibility of the *C. parapsilosis* complex and *C. guilliermondii* involve the occurrence of polymorphisms in hot spot 1 of the Fks1 subunit, although the clinical significance is uncertain as these species are often successfully treated with echinocandins (Perlin, 2015; Dudiuk et al., 2017).

It is known that *fks*1 mutations can result in different therapeutic responses to different echinocandins and may or may not be associated with reduced virulence and fitness (Lackner et al., 2014; Perlin, 2015). Reduced virulence was previously related to altered kinetics of glucan synthase, resulting in changes in cell wall composition and decreased transmission between patients of strains harboring these mutations (Perlin, 2015). However, fitness may depend not only on the nature and position of the substitution but also on host immunity, as significant differences were found between immunocompetent and neutropenic hosts (Lackner et al., 2014).

Aside from the known consequences of *fks*1 mutations in echinocandin resistance, the functions of the Fks2 and Fks3 subunits are not yet entirely elucidated and may differ among species. Although echinocandin resistance in *S. cerevisiae* is conferred by *fks*2 mutations, both *fks*2 and *fks*3 deletion in this species resulted in better growth under exposure to caspofungin (Suwunnakorn et al., 2018). Moreover, a recent study demonstrated that Fks2 and Fks3 are not functionally redundant in *C. albicans* and suggested the action of both subunits as negative regulators of Fks1 (Suwunnakorn et al., 2018).

The *in vitro* efficacy of echinocandin drugs against dermatophytes was firstly evaluated by Bao et al. (2013), who demonstrated that caspofungin and micafungin did not entirely inhibit growth, but exhibit good *in vitro* antifungal activity to dermatophytes. Recent studies also showed that anidulafungin has potent *in vitro* activity against dermatophytes although the relevance for clinical efficacy has not yet been established (Badali et al., 2015; Baghi et al., 2016; Deng et al., 2017). The absence of *in vivo* studies of echinocandins efficacy limits its use for treatment of dermatophytosis, which may underlie the absence of resistant dermatophyte strains described to date.

### Structural Elements Influencing Drug Resistance

In addition to mechanisms involving stress and alteration of the target drug, other factors such as the fungal structure and cellular organization can also interfere with drug resistance/tolerance. For example, biofilms are comprised of complex, surface-associated cell populations embedded in an extracellular matrix and are considered the most prevalent forms of microbial growth. They are responsible for a broad spectrum of microbial infections in humans and are associated with increased resistance to antifungal therapy. Factors that contribute to biofilm drug resistance include structural complexity, presence of extracellular matrix, metabolic heterogeneity, and up-regulation of efflux pump genes. The matrix that surrounds the microbial population acts as a physical barrier, protecting the microbial community against drugs and host immune response and contributing to the emergence of persistent cells, a subpopulation able to tolerate higher concentrations of drugs and considered to enable recalcitrance (Fanning and Mitchell, 2012; Robbins et al., 2017). As recalcitrance is often associated with dermatophytosis, it has been proposed that biofilms may act as a source of persistent infection and antifungal resistance in onychomycosis (Burkhart et al., 2002; Gupta A.K. et al., 2016). Consistent with this concept, dermatophytes have been shown to form biofilms *in vitro* (Costa-Orlandi et al., 2014). Recently, the formation of biofilms in nail fragments has also been demonstrated in an *in vitro* model of onychomycosis (Brilhante et al., 2017).

The production of arthroconidia by dermatophytes has also been proposed to contribute to drug resistance. Arthroconidia are produced by the fragmentation of hyphae and are considered as the main mode of transmission of dermatophytosis (Gupta and Kohli, 2003). Arthroconidia are more resistant to antifungal drugs than hyphae (Arrese et al., 2001) and, in general, are more resistant than microconidia as well (Coelho et al., 2008).

## Conclusion and Perspectives

The ideal antifungal agent has been postulated as the compound that inhibits potential virulence factors, affects fungi physiology, and also acts on specific fungal cellular targets. Recently, the genomes of about twenty dermatophyte strains were sequenced through the Broad Institute initiative, and the databases are available at Broad Fungal FTP Site^1^. Consequently, advances in dermatophyte research have been reported, improving the understanding of their pathophysiology, niche and host preferences, and general aspects of gene structure and regulation, which together strongly contribute to the development of strategies for more effective therapies (Martinez-Rossi et al., 2017). Additionally, the comparative genome analysis highlighted the kinases as an enriched class in dermatophyte genomes, reemphasizing the interplay between signal transduction and regulation with dermatophyte–host interaction (Martinez et al., 2012). Some such kinases, with unknown specificities, may be associated with intrinsic abilities of dermatophytes to cause disease. Here, we discussed the modulation of kinase-encoding genes in response to antifungal drugs along with their putative roles in drug-stress responses. Furthermore, the current studies shed light on potential novel antifungal targets such as the glyoxylate cycle, which is absent in mammalian cells and plays an adaptive role during infection (Lorenz and Fink, 2001). Another interesting target is FAS, owing to the marked architectural differences between fungal and mammalian FAS proteins, and to the importance of fatty acid biosynthesis for fungal physiology (Maier et al., 2010). Additionally, Hsp90 has been considered as a therapeutic target to treat fungal infections, as it is essential for cell physiology and participates in the response to several stresses including the exposure to antifungals as well as in fungal–host interactions (Jacob et al., 2015; Martinez-Rossi et al., 2016, 2017). Together, these potential targets play important roles in fungal adaptation, physiology, and the infection process. In this respect, both synthetic and natural compounds constitute promising sources of new structural entities with antifungal properties that exhibit potentially novel mechanisms of action. Moreover, these natural compounds may be administrated in association with conventional antifungal agents, synergistically enhancing their antifungal activity and acting as chemosensitizers to overcome fungal resistance. Therefore, the natural sources may expand the possibilities to improve or develop new and effective antifungal therapies.

## Author Contributions

All authors reviewed literature and wrote sections of the manuscript. NM-R, TB, NP, EL, and AR edited the manuscript. TB and NQ designed the figure. TB, NP, and EG prepared the tables. All authors read and approved the submitted version.

## Conflict of Interest Statement

The authors declare that the research was conducted in the absence of any commercial or financial relationships that could be construed as a potential conflict of interest.

## Footnotes

1 ftp://ftp.broadinstitute.org/pub/annotation/fungi
